# Design and methods for a quasi-experimental pilot study to evaluate the impact of dual active ingredient insecticide-treated nets on malaria burden in five regions in sub-Saharan Africa

**DOI:** 10.1186/s12936-021-04026-0

**Published:** 2022-01-10

**Authors:** Adama Gansané, Baltazar Candrinho, Aimable Mbituyumuremyi, Perpetua Uhomoibhi, Sagnon NFalé, Audu Bala Mohammed, Wamdaogo Moussa Guelbeogo, Antoine Sanou, David Kangoye, Siaka Debe, Moubassira Kagone, Emmanuel Hakizimana, Aline Uwimana, Albert Tuyishime, Chantal M. Ingabire, Joseph H. Singirankabo, Hannah Koenker, Dulcisaria Marrenjo, Ana Paula Abilio, Crizologo Salvador, Binete Savaio, Okefu Oyale Okoko, Ibrahim Maikore, Emmanuel Obi, Samson Taiwo Awolola, Adedapo Adeogun, Dele Babarinde, Onoja Ali, Federica Guglielmo, Joshua Yukich, Sara Scates, Ellie Sherrard-Smith, Thomas Churcher, Christen Fornadel, Jenny Shannon, Nami Kawakyu, Emily Beylerian, Peder Digre, Kenzie Tynuv, Christelle Gogue, Julia Mwesigwa, Joseph Wagman, Monsuru Adeleke, Ande Taiwo Adeolu, Molly Robertson

**Affiliations:** 1 Centre National de Recherche et de Formation sur le Paludisme, Ouagadougou, Burkina Faso; 2 National Malaria Control Programme, Ministry of Health, Maputo, Mozambique; 3 Rwanda Biomedical Centre, Ministry of Health, Kigali, Rwanda; 4 National Malaria Elimination Programme, Federal Ministry of Health, Abuja, Nigeria; 5 University of Rwanda, Kigali, Rwanda; 6 Tropical Health LLP, London, UK; 7 National Institute of Health, Maputo, Mozambique; 8 PATH, Maputo, Mozambique; 9 Nigerian Institute of Medical Research, Lagos, Nigeria; 10 Ibolda Health International, Abuja, Nigeria; 11 Liverpool School of Tropical Medicine, Liverpool, UK; 12 School of Public Health and Tropical Medicine, Tulane University, New Orleans, LA USA; 13 MRC Centre for Global Infectious Disease Analysis, School of Public Health, Imperial College London, London, UK; 14 IVCC, Liverpool, UK; 15 PATH, Seattle, WA USA; 16 PATH, Washington, DC USA; 17 PATH, Kampala, Uganda; 18 Osun State University, Osun State, Osogbo, Nigeria; 19 University of Ilorin, Kwara State, Ilorin, Nigeria

**Keywords:** Malaria, Vector control, Dual-AI ITNs, Burkina Faso, Mozambique, Nigeria, Rwanda

## Abstract

**Background:**

Vector control tools have contributed significantly to a reduction in malaria burden since 2000, primarily through insecticidal-treated bed nets (ITNs) and indoor residual spraying. In the face of increasing insecticide resistance in key malaria vector species, global progress in malaria control has stalled. Innovative tools, such as dual active ingredient (dual-AI) ITNs that are effective at killing insecticide-resistant mosquitoes have recently been introduced. However, large-scale uptake has been slow for several reasons, including higher costs and limited evidence on their incremental effectiveness and cost-effectiveness. The present report describes the design of several observational studies aimed to determine the effectiveness and cost-effectiveness of dual-AI ITNs, compared to standard pyrethroid-only ITNs, at reducing malaria transmission across a variety of transmission settings.

**Methods:**

Observational pilot studies are ongoing in Burkina Faso, Mozambique, Nigeria, and Rwanda, leveraging dual-AI ITN rollouts nested within the 2019 and 2020 mass distribution campaigns in each country. Enhanced surveillance occurring in select study districts include annual cross-sectional surveys during peak transmission seasons, monthly entomological surveillance, passive case detection using routine health facility surveillance systems, and studies on human behaviour and ITN use patterns. Data will compare changes in malaria transmission and disease burden in districts receiving dual-AI ITNs to similar districts receiving standard pyrethroid-only ITNs over three years. The costs of net distribution will be calculated using the provider perspective including financial and economic costs, and a cost-effectiveness analysis will assess incremental cost-effectiveness ratios for Interceptor® G2, Royal Guard®, and piperonyl butoxide ITNs in comparison to standard pyrethroid-only ITNs, based on incidence rate ratios calculated from routine data.

**Conclusions:**

Evidence of the effectiveness and cost-effectiveness of the dual-AI ITNs from these pilot studies will complement evidence from two contemporary cluster randomized control trials, one in Benin and one in Tanzania, to provide key information to malaria control programmes, policymakers, and donors to help guide decision-making and planning for local malaria control and elimination strategies. Understanding the breadth of contexts where these dual-AI ITNs are most effective and collecting robust information on factors influencing comparative effectiveness could improve uptake and availability and help maximize their impact.

**Supplementary Information:**

The online version contains supplementary material available at 10.1186/s12936-021-04026-0.

## Background

The World Health Organization (WHO) estimated that in 2019, 229 million cases of malaria occurred globally, resulting in 409,000 deaths [1]. While this is a remarkable improvement in since 2000, the global downward trend in annual malaria incidence began to plateau in 2014 and, more recently, the COVID-19 pandemic has complicated future projections. Challenges in keeping hard-won gains, and in accelerating progress toward elimination, have led the WHO to describe the global fight against malaria as at a crossroads, calling for increased funding and to develop, optimize, and implement urgently needed new tools and approaches [2].

Attaining optimal coverage of populations at risk of malaria with vector control interventions—primarily insecticide-treated bed nets (ITNs) and indoor residual spraying (IRS)—remains a priority because of its importance in malaria control and elimination [3, 4]. Per WHO, optimal coverage refers to (1) providing populations at risk of malaria with access to ITNs coupled with health promotion to maximize use and (2) ensuring timely replacement; or providing these populations with the regular application of IRS. Either intervention should be deployed at a level that provides the best value for money while reflecting programmatic realities [5]. Unfortunately, the continued effectiveness of these interventions is threatened by the spread of pyrethroid resistance in most endemic countries [1], posing a significant risk to the sustained impact of these tools [2, 3]. Evidence shows that standard pyrethroid-only ITNs (which here are synonymous with traditional long-lasting insecticidal nets, or LLINs) can still be effective at preventing malaria in regions with resistant vector populations [6]; however, as evidence of pyrethroid resistance mounts and documentation of the decreased ability of standard pyrethroid-only ITNs to control resistant mosquito populations increases, new classes of ITNs have been developed to perform against pyrethroid-resistant mosquitoes. These include pyrethroid-based ITNs that contain the insecticide synergist piperonyl butoxide (PBO, which inhibits mosquito monooxygenase enzymes that can detoxify pyrethroids), as well as newer dual active ingredient (dual-AI) ITNs that combine a pyrethroid AI with a second from a different class of insecticide—most notably either the pyrrole chlorfenapyr, an insecticide that disrupts adenosine triphosphate synthesis, or the insect growth regulator pyriproxyfen, which disrupts mosquito development and reproduction. ITNs refers to all bed nets treated with an insecticide and/or an insecticide synergist. The WHO defines long-lasting insecticidal nets as bed nets that have proven effectiveness after 20 washes and three years of use [7]. The dual active ingredient and piperonyl butoxide ITNs evaluated in this study have not demonstrated that they meet this standard.

Experimental hut and cluster randomized control trials suggest that these dual-AI ITN types provide greater mosquito control and superior protective efficacy against various epidemiological and entomological outcomes in areas with pyrethroid-resistant vectors [8]. For example, in Burkina Faso the distribution of ITNs with permethrin and pyriproxyfen (Olyset® Duo; Sumitomo Chemical Co., Ltd.) reduced clinical malaria incidence in children under 5 years old by 12% and infective mosquito bites by 51% compared to the standard pyrethroid-only ITNs [9]. In Tanzania, compared to standard pyrethroid-only ITNs, ITNs with PBO plus permethrin (Olyset® Plus, Sumitomo Chemical Co., Ltd) provided 44% greater reduction in prevalence at 9 months and 33% greater reduction at 21 months [10]. ITNs with alpha-cypermethrin and chlorfenapyr have shown high efficacy at controlling pyrethroid-resistant mosquitoes in experimental hut studies [11–13], demonstrating potential to better control malaria across sub-Saharan Africa. ITNs with alpha-cypermethrin and the insect growth regulator pyriproxyfen were also found to have high efficacy in controlling pyrethroid-resistant mosquitoes through feeding inhibition, sterilization, and the induction of mortality during experimental hut studies [14].

Access to new dual-AI ITNs is challenged by limited efficacy data for policy recommendations, higher costs than standard pyrethroid-only ITNs, and lack of evidence on cost-effectiveness compared with standard pyrethroid-only ITNs. However, in Phase 1 and 2 testing where safety and entomological impacts are assessed, Interceptor G2® ITNs (IG2; BASF AG), which combine alpha-cypermethrin and chlorfenapyr, performed to the thresholds required of pyrethroid-only ITNs and had no known adverse effects, receiving conditional WHO prequalification in January 2018 [15]. Similarly, Royal Guard® (RG; Disease Control Technologies, LLC), which uses a combination of alpha-cypermethrin and pyriproxyfen, received WHO prequalification in March 2019 [16]. While IG2 and RG ITNs have been approved for conditional use, WHO Vector Control Advisory Group guidance states that further evidence around dual-AI ITNs, specifically demonstration of their incremental epidemiological impact and the durability of this impact, is needed before policy recommendations can be made for their preferential use against pyrethroid resistant mosquitos (Additional file 1: Table S1].

The New Nets Project (NNP), funded by The Global Fund to Fight AIDS, Tuberculosis and Malaria and Unitaid, aims to help expand the evidence base for rational use of IG2, RG, and other ITNs with novel insecticide formulations while expediating the distribution of these nets to communities. This study focuses on evaluating the pilot distributions of IG2 taking place in Burkina Faso, Mozambique, Nigeria, and Rwanda, and RG in Mozambique and Nigeria. In addition, durability monitoring data will be collected by either NNP partners or by the US President’s Malaria Initiative (PMI)–funded VectorLink Project, and costing data for each country will be collected by NNP partners.

## Study objectives

The overall aim of the study is to describe the effectiveness and cost-effectiveness of IG2, RG, and PBO ITNs in comparison to standard pyrethroid-only ITNs. Specific objectives are organized into five study components: (1) malaria epidemiological surveillance to evaluate the impact of dual-AI ITNson malaria burden, (2) entomological surveillance to evaluate the impact of dual-AI ITNs on key entomological outcomes (3) anthropological data to assess how transmission risk is influenced by human behaviours, (4) assessment of the chemical and physical durability of the new ITN types, and (5) evaluation of the cost and cost-effectiveness of dual-AI ITNs.

1. The epidemiological objective is to evaluate the effectiveness of IG2, RG, and PBO ITN distributions, compared to standard pyrethroid-only ITN mass distributions, in reducing:
  - Malaria prevalence measured during annual cross-sectional surveys in children 6 months to 5 years (Burkina Faso, Mozambique, and Nigeria) and all ages (Rwanda).
  - Passive malaria case incidence rates measured using routine surveillance systems (DHIS2).
2. The entomological objective is to compare post-ITN distribution trends in vector densities, human biting rates, and entomological inoculation rates using CDCLTs and HLCs and to establish baseline. In addition, entomological objective is to evaluate insecticide susceptibility patterns in the primary vector species populations.
3. The anthropological objective is to assess how human behaviour influences exposure risk and understand the key drivers of ITN use and non-use behaviour across districts with different types of ITN types.
4. The ITN durability monitoring objective is to assess the attrition, survivorship, physical integrity, and insecticidal bioefficacy of all ITN types at baseline, 12 months, and 24 months.
5. The cost-effectiveness objective is to combine data on product effectiveness (based upon case incidence rates measured for objective 1) and data on product prices and costs of delivery and deployment to estimate a mean cost per case averted for each ITN type.

## Methods

### Study sites

The study is composed of five distinct evaluations across four countries, one each in Burkina Faso, Nigeria, and Rwanda and two in Mozambique (Table 1). In each evaluation setting, these observational pilot studies accompany the national malaria control programme (NMCP)-led mass distribution campaigns of 2019 and 2020.

**Table 1:** Study districts, type of insecticide-treated bed nets distributed through the mass campaign, and the pre-study baseline prevalence

Country	Study area	ITN AI	ITN brand	Under 5 baseline prevalence* (%)	Primary vector species	Pyrethroid resistance status (WHO tube test mortality)
Burkina Faso [17]	Banfora	Chlorfenapyr + alpha-cypermethrin	Interceptor G2	RDT: 13.8	*An. gambiae*	High (< 50%)
	Gaoua^†^	Standard pyrethroid-only	DuraNet LN, Interceptor, MAGNet, PermaNet 2.0	RDT: 32.4	*An. gambiae*	High (< 50%)
	Orodara	PBO	PermaNet 3.0	RDT: 13.6	*An. gambiae*	High (< 50%)
Northern Mozambique [18]	Mandimba	Pyriproxyfen + alpha-cypermethrin	Royal Guard	RDT: 48.6	*An. funestus*	Moderate (85–100%)
	Cuamba	Chlorfenapyr + alpha-cypermethrin	Interceptor G2	RDT: 48.6	*An. funestus*	Moderate (85–100%)
	Gurue^†^	Standard pyrethroid-only	DuraNet LN	RDT: 44.3	*An. funestus*	Moderate (85–100%)
Western Mozambique [18]	Changara	PBO	Olyset Plus	RDT: 29.4	*An. gambiae*	High (60–85%)
	Guro	Chlorfenapyr + alpha-cypermethrin	Interceptor G2	RDT: 47.6	*An. gambiae*	High (60–85%)
	Chemba^†^	Standard pyrethroid-only	DuraNet LN	RDT: 29.4	*An. gambiae*	High (60–85%)
Nigeria [19]	Asa	Chlorfenapyr + alpha-cypermethrin	Interceptor G2	RDT: 43.7	*An. gambiae*	Variable (12–94%)
	Moro	Pyriproxyfen + alpha-cypermethrin	Royal Guard	RDT: 43.7	*An. gambiae*	Variable (12–94%)
	Ejigbo^†^	Standard pyrethroid-only	DuraNet LN	RDT: 54.9	*An. gambiae*	Variable (12–94%)
	Ife North	Alpha-cypermethrin + PBO	Veeralin	RDT: 54.9%	*An. gambiae*	Variable (12–94%)
Rwanda [20]	Karongi	Chlorfenapyr + alpha-cypermethrin	Interceptor G2	RDT: 3.1 Microscopy: 1.8	*An. funestus*	Low (85–100%)
	Nyamagabe^†^	Deltamethrin	Yahe LN	RDT: 14.4 Microscopy: 8.7	*An. funestus*	Low (85–100%)
	Ruhango	Deltamethrin (and IRS)	Yahe LN	RDT: 14.4 Microscopy: 8.7	*An. funestus*	Low (85–100%)

*IG2* Interceptor G2, *IRS* indoor residual spraying, *ITN* insecticide-treated bed net, *PBO* piperonyl butoxide, *RDT* rapid diagnostic test, *RG* Royal Guard

*Prevalence surveys were not conducted at the study area level. Details on each survey methodology can be found in the referenced document for each country. Additional details on each evaluation context are available in Additional file 2: Annex S1

^†^Indicates study control area

**Fig. 1:**
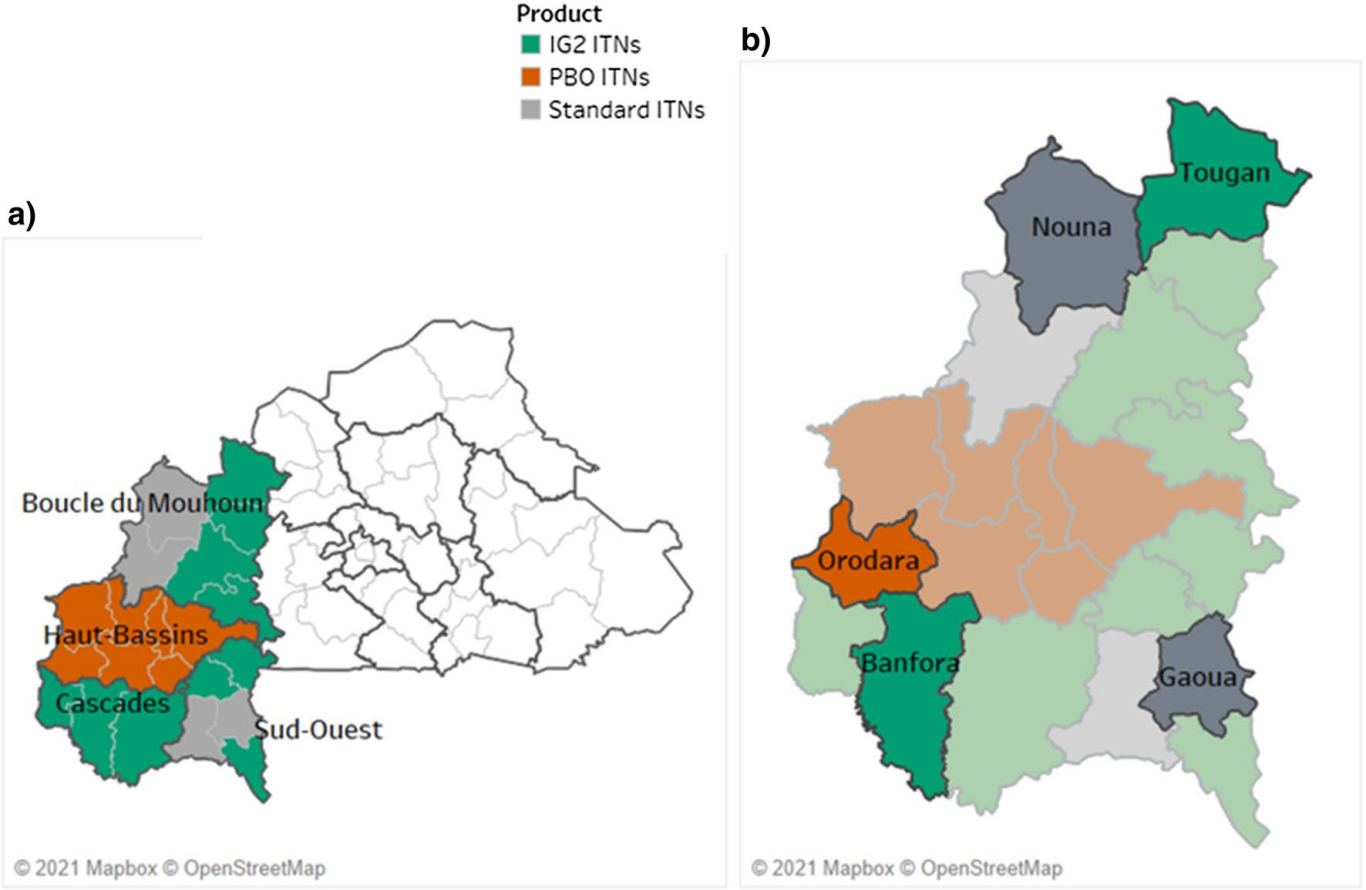
Study districts in Burkina Faso

**Fig. 2:**
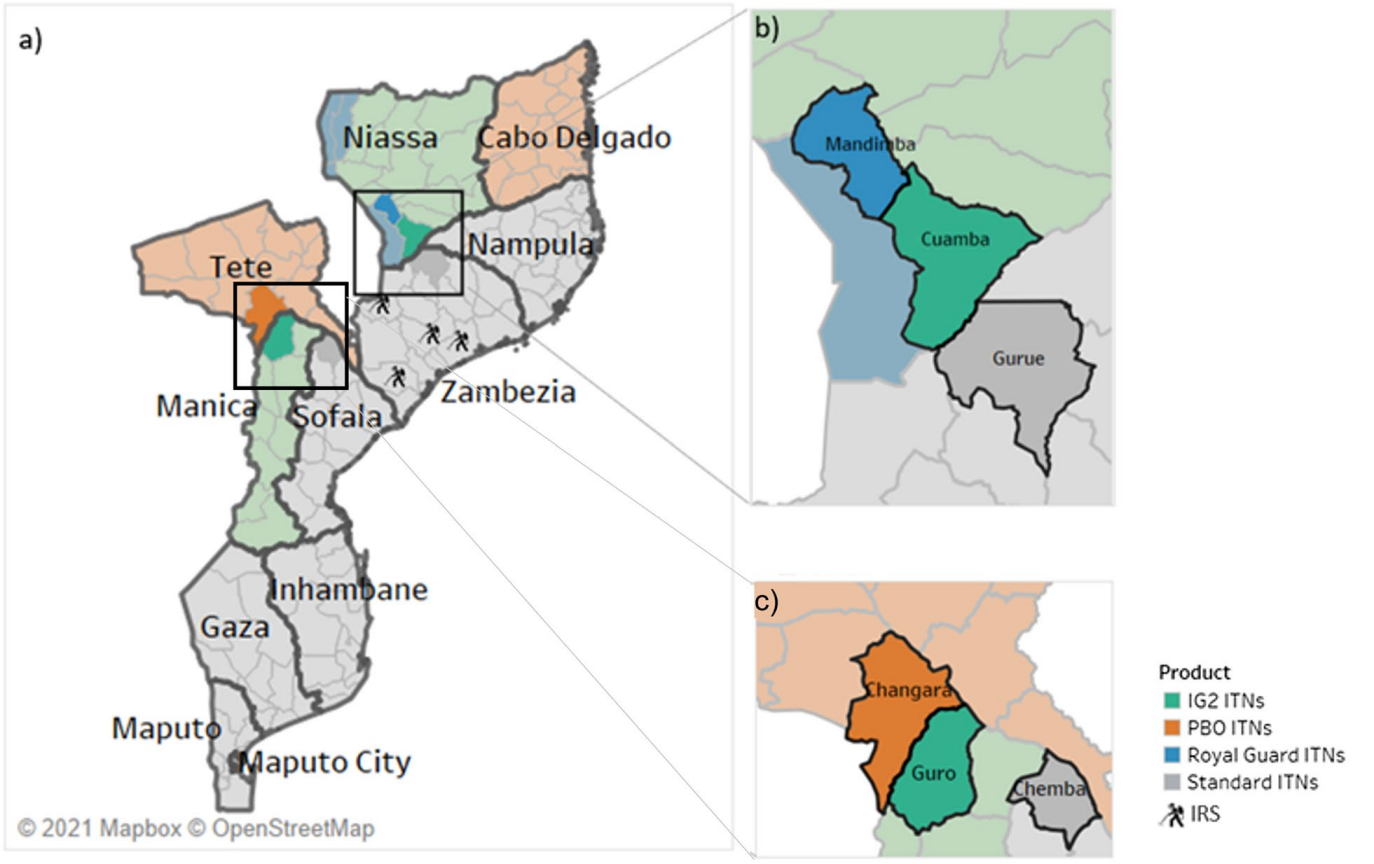
Study districts in Mozambique

**Fig. 3:**
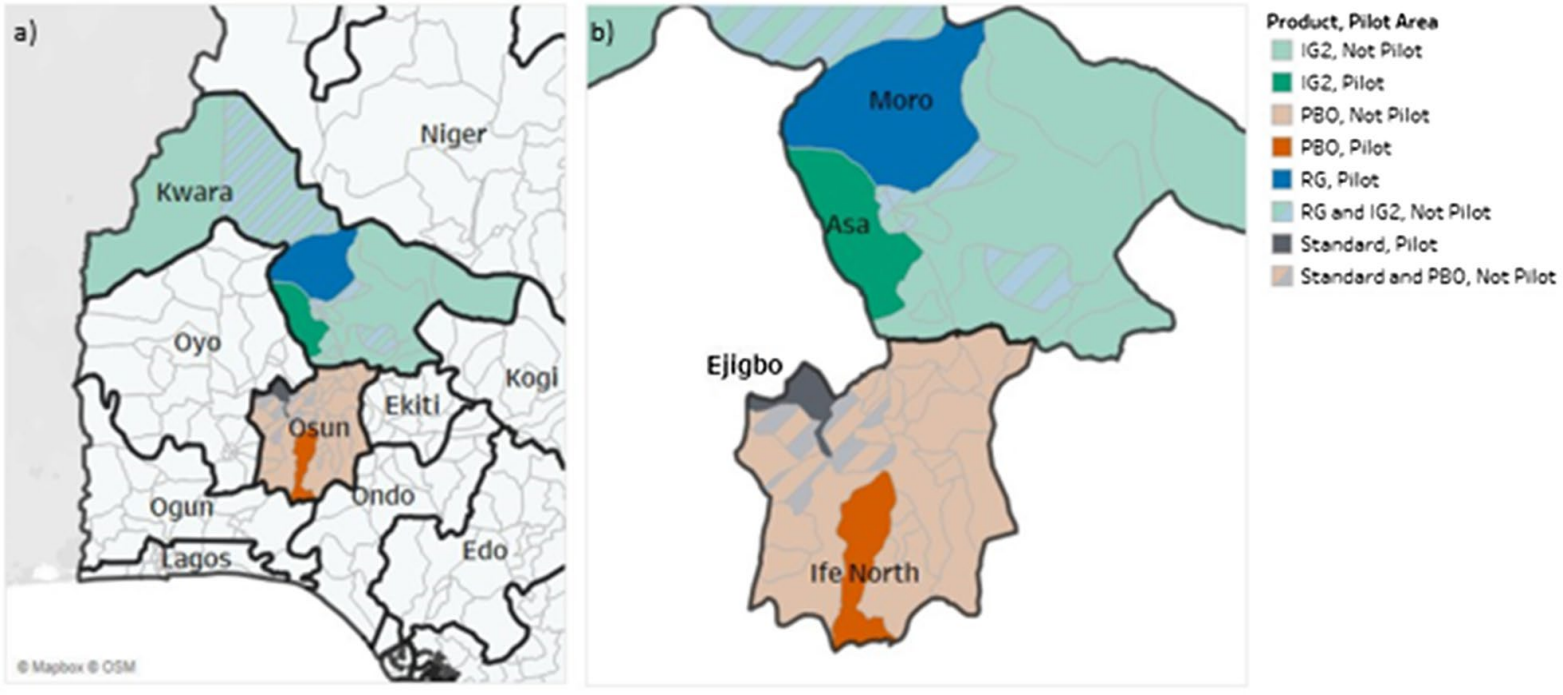
Study districts in Nigeria

**Fig. 4:**
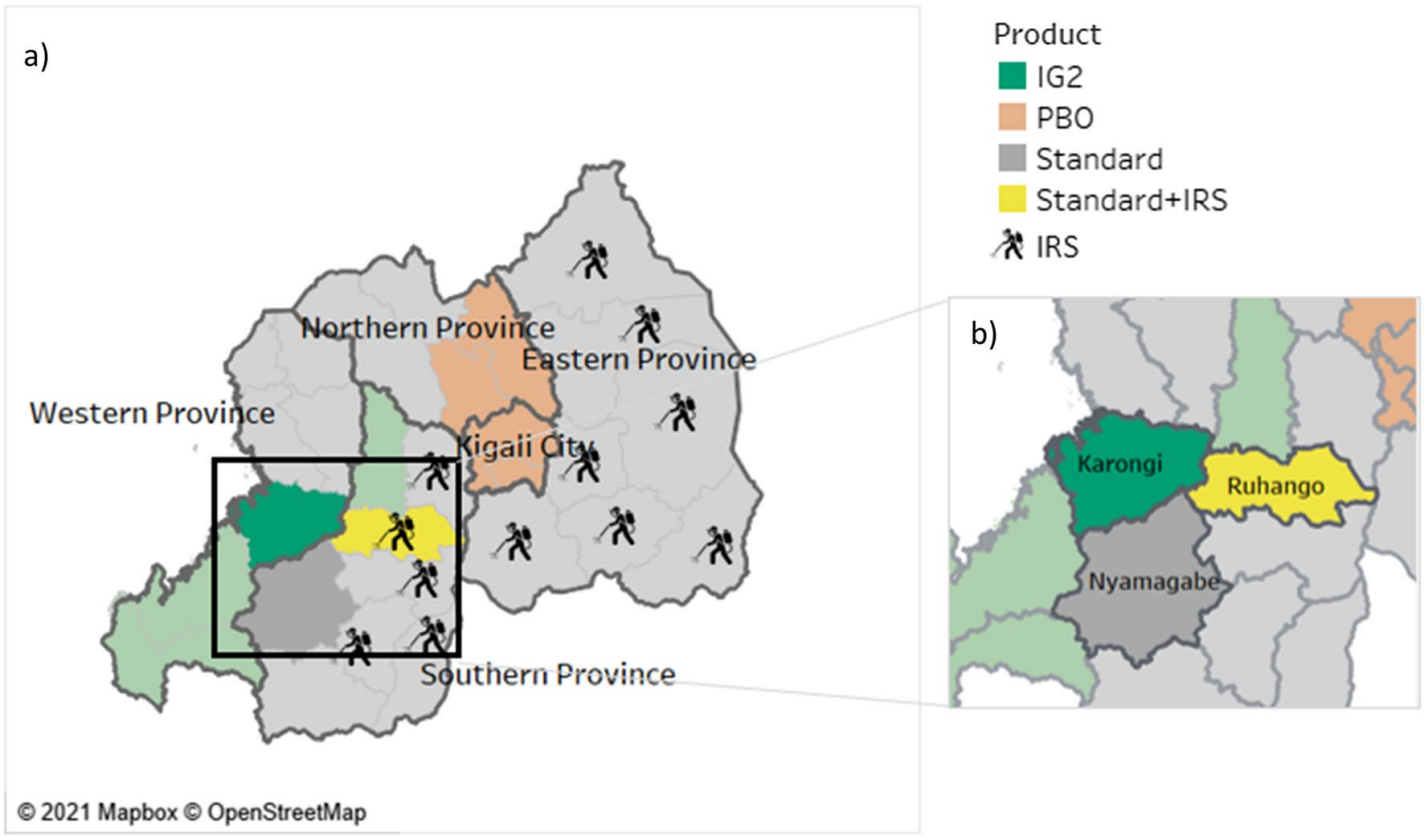
Study districts in Rwanda

IG2s were deployed in five districts (Burkina Faso, Nigeria, western and northern Mozambique, Rwanda), RGs deployed in two districts (Northern Mozambique and Nigeria) and PBOs deployed in three districts (Burkina Faso, Western Mozambique, and Nigeria). In Rwanda, one district deployed IRS in combination with standard pyrethroid-only ITNs in comparison to pyrethroid-only ITNs alone (Figs. 1, 2, 3 and 4).

### Study design

The study is an opportunistic, quasi-experimental design with control groups; dual-AI ITNs (intervention) were distributed alongside standard pyrethroid-only ITNs (control) during multi-product universal coverage campaigns. As such, these are all observational studies that leverage the 2019 and 2020 ITN mass distribution campaigns and the district health information systems and existing vector surveillance programmes, which serve as monitoring platforms. Intervention districts received a dual-AI ITN type (IG2, RG, or PBO), while control districts received either standard pyrethroid-only ITNs or standard pyrethroid-only ITNs plus IRS. The ITN types are not always distinguishable by size, shape, or colour, but are identifiable by their sewn-in product labels.

### Identification of intervention and control study districts

Decisions on what brand/type of ITN to procure, and where to distribute them were made by each respective NMCP through consultation with partner institutions and international donors. The NMCPs used the dual-AI ITNs in their mass campaigns with the stipulation that there be an accompanying pilot evaluation based on their distribution and the study designs took advantage of natural comparisons among districts that results from these decisions. The NMCPs of Burkina Faso (2019) and Rwanda (2020) incorporated IG2 ITNs into their mass distribution campaigns. Mozambique and Nigeria both incorporated IG2 and RG ITNs into their 2020 mass campaigns. In addition, Burkina Faso (2019), Mozambique (2020), and Nigeria (2020) incorporated PBO ITNs into their mass campaigns. All districts included in the mass distribution campaigns receive a single type of net. A subset of districts that receive IG2, RG, PBO, and pyrethroid-only ITNs were selected by the partners as study districts (Table 2).

**Table 2:** ITN distribution by country National Malaria Control Programmes

Geography	Study period	ITNs evaluated	ITN distribution completed
Burkina Faso	2019–2022	IG2, PBO	June 2019 (PBO) August 2019 (standard) October 2019 (IG2)
Northern Mozambique	2020–2022	IG2, RG	November 2020
Western Mozambique	2020–2022	IG2, PBO	December 2020
Rwanda	2020–2022	IG2	February 2020 (standard) May 2020 (IG2)
Nigeria	2020–2022	IG2, RG, PBO	November 2020

### Epidemiological component

Across all countries, malaria burden is measured through cross-sectional surveys, routine health facility data, and in most evaluations, antenatal care surveillance. For all countries, the study aims to conduct all surveys during high transmission seasons. This is complicated in many areas because of seasonal rains that impact operations. In addition, because of the timing of the grant and the net distribution, with the necessity to conduct a baseline survey before or contiguous with distribution, the timing of baseline surveys were adjusted in Rwanda, Nigeria, and Mozambique. Efforts are made to ensure that the post distribution cross-sectional survey timing is within a higher transmission season and is consistent across the years for comparison. In Burkina Faso, where four rounds of SMC are done annually in children 3 months to 5 years during the malaria transmission season, the study aims to time the surveys before SMC campaigns to ensure that the measurements are not conflated with the effects of the first round of seasonal malaria chemoprevention.

In each study district, villages were randomly selected for participation using probability proportionate to size, from which households were sampled either randomly or following a standard systematic sampling approach (e.g., every third household) During each survey a new set of households with eligible participants are selected (Table 3).

**Table 3:** Cross-sectional survey methodology by country

Country	Participants		Sample size (households)	Survey months
	Questionnaire	Prevalence		
Burkina Faso	Household head or primary caregiver	Child aged 6 to 59 months	570 per year (2280 total)	• July 2019 • July 2020 • July 2021 • July 2022
Northern and western Mozambique	Household head or primary caregiver	Child aged 6 to 59 months	2520 per year (7,560 total)	• September–October 2020 • September–October 2021 • August–September 2022
Nigeria	Household head or primary caregiver	Child aged 6 to 59 months	1680 per year (5,040 total)	• September 2020 • November 2020 • November 2021
Rwanda	Household head or primary caregiver	All household residents aged 6 months and older	450 per year (1,800 total)	• February 2020 • December 2020 • November 2021 • November 2022

*LGA* local government area

### Eligibility for cross-sectional surveys

Households were eligible for inclusion in the survey if they had at least one child 6 months to 59 months. If a household had more than one eligible child at the time of the survey, the children’s names are listed in chronological birth order and a random number list is used to select one child for participation. In Rwanda, all household members of all ages are enrolled for the survey and were tested. The decision to enroll all ages from all households was based on the NMCPs desire to assess infection across all ages.

### Inclusion

The inclusion criteria for cross-sectional surveys are as follows:

1. Confirmed residency in the village.
2. Households with eligible children within the target age group for each country.
3. Written informed consent: primary caregivers will provide consent for minors; in addition to primary caregiver consent, assent will be obtained as appropriate in each country; adults will provide informed consent for themselves.

### Exclusion

The exclusion criteria for cross-sectional surveys are as follows:

- Households with no children within the target age group will be excluded for each country.
- Households or individuals who did not provide informed consent or assent.
- Households with children in the age group but are not resident in the village for at least six months.

Participants present at the time of the house visit are tested for malaria by conventional malaria rapid diagnostic test (RDT) and those with a positive RDT are treated following national malaria treatment guidelines [21–24]. A questionnaire is administered to each head of house or representative to collect data on demographic characteristics of household members, house structure, socio-economic variables, care-seeking behaviour and ITN indicators including the ITNs per house, ITN ownership and utilization at night.

Passive surveillance of malaria cases comes from routine health facility data collected during outpatient health facility visits or from community health workers in each study district. Analysis of routine malaria health management information system data starts approximately one year prior to ITN campaigns. In addition, in Nigeria, Western Mozambique, and Burkina Faso, data on the prevalence of malaria infection is assessed from pregnant women attending their first antenatal care visits in selected health facilities. Data quality assessments are conducted in some countries to assess the quality and completeness of malaria surveillance data. Table 3 details the similarities and differences in cross-sectional survey methods used in each country.

### Entomological component

A core set of collection methods are undertaken for the entomological surveillance component of the study, including human-baited US Centers for Disease Control and Prevention light traps (CDCLTs), human landing collections (HLCs), and larval sampling, supplemented by pyrethrum spray catches in Nigeria where HLCs are not performed (Table 4). Entomological investigations are nested within routine NMCP vector surveillance programme activities as much as possible so that within each district, operational sentinel villages with existing entomological surveillance capacity are enhanced to support (1) increased sampling from additional houses and (2) molecular characterization of vector species collected. In each county, pre-existing entomological sentinel sites had been established on the basis of historical mosquito densities, ease of access, and safety. They do not necessarily overlap with the same communities participating in the household surveys, which are selected randomly.

**Table 4:** Entomological methods by country

		Burkina Faso	Northern and Western Mozambique	Nigeria	Rwanda
CDCLT collections	Number of collections per district	• 3 villages with 6 houses each		• 4 villages with 2 houses per village • Paired indoor-outdoor collections	• 9 villages with 5 houses per village
	Frequency	• Once per week • Two consecutive nights		• Three consecutive nights • Once a month	• Two consecutive nights • Twice per month
HLCs	Number of collections per district	• 3 villages with 2 houses each • Paired indoor-outdoor collections	• 3 villages with 3 houses each • Paired indoor-outdoor collections		• 3 houses per village • Paired indoor-outdoor collections
	Frequency	• Concurrent with CDCLT collections (different houses)	• Two consecutive nights • Every other month for 24 months		• Concurrent with CDCLT collections (different houses)
Pyrethrum spray catches	Number of collections per district			• 48 houses	
	Frequency			• Once monthly	
Larval sampling	Frequency	• Surveys at least once per year	• Surveys at least once per year (aligned with standard NMCP surveillance)	• Surveys at least once per year	• Surveys at least once per year

*CDCLT* US Centers for Disease Control and Prevention light trap, *HLC* human landing collection, *LGA* local government area

#### CDC light traps

CDCLTs monitor host–seeking *Anopheles* mosquito distribution and density. Traps are set and hung at the foot of one household sleeping space in which the sleepers are protected by a bed net (from approximately one hour before sunset until one hour after sunrise), following standard WHO vector surveillance procedures [25]. Entomological sentinel sites have been chosen based on historical mosquito densities, ease of access, and safety. In each sentinel site, village leadership is engaged to help orient the community to entomological surveillance methods and to identify households for participation through a systematic sampling approach: typically, every third household is visited until the target number of households have been obtained. In Nigeria, outdoor human-baited CDCLTs are utilized, placed about 5 to 10 m from the entrance of each sentinel house where indoor CDCLT collections are also occurring.

#### Human landing collections

HLCs assess vector densities, human biting rates and malaria transmission by estimated entomological inoculation rates [25]. Paired collectors are located with one indoors (within 1 to 5 m of the entrance to the home) and one in the outdoor peri-domestic environment approximately 5 to 10 m from the household entrance. HLCs occur over 12 h, approximately one hour before sunset until approximately one hour after sunrise, with paired collectors alternating locations regularly. As with CDCLT collections, village leadership is engaged to help orient the community to entomological surveillance methods and to identify households for participation through a systematic sampling approach, though current COVID-19 mitigation protocols for HLC prioritize the collection of mosquitoes from within the collectors’ own households or households of family members.

#### Anopheline species identification

All *Anopheles* mosquitoes sampled during CDCLT and HLC collections are identified morphologically to species group [26] and stored with silica gel for molecular processing and polymerase chain reaction analyses, including species identification [27, 28], parasite screening [29], and measurement of *kdr* [30] and ace-1 mutation frequencies [31], as appropriate. A subsample of specimens from the primary vector species populations collected during HLC is also assessed for age using standard parity dissection methods [25].

#### Larval sampling and insecticide resistance testing

Larval sampling, following WHO Global Malaria Programme recommendations [25], is conducted annually in each district/LGA during times of peak adult vector density. Specimens from various known larval habitats within the above sentinel villages are combined to provide a sufficient population of test mosquitoes from each district for appropriate bioassay testing. As possible, test mosquito populations arise from multiple F_0_ oviposition events from various locations, with the goal that the test mosquitoes reflect a range of genotypic and phenotypic resistance traits from the target population. Test populations of *Anopheles funestus* mosquitoes are likely to develop from adult female resting collections, similarly pooling specimens collected from multiple locations within each district. *Anopheles* mosquito densities dictate the level of testing performed; at a minimum, this will include species-specific pyrethroid resistance frequency and PBO synergist assays using WHO tube bioassays; and may also include CDC bottle bioassays to characterize pyrethroid insecticide resistance intensity and additional tunnel and cone tests to determine susceptibility to chlorfenapyr and pyriproxyfen, as appropriate.

#### Pyrethrum spray catches

In Nigeria, standardized pyrethrum spray catches are used to sample the indoor resting populations of mosquitoes in 48 households in each of the four local government areas. Existing sentinel houses used for entomological surveillance, which had been purposefully selected to be representative of local diversity in both village geography and housing structure, are used. If additional surveillance is needed, houses will be randomly selected from each village. If consent is withdrawn for any reason, the house is replaced with one matched for location and building material.

### Anthropological component

The anthropological data center on two main areas: (1) understanding of transmission risk, and (2) understanding ITN use and behaviour using a mixed-methods approach with a quantitative component nested within a broader qualitative component. The quantitative component assesses transmission risk by measuring the actual time individuals are unprotected (not under an ITN) and when they are protected (under an ITN). The collection sites are paired with the entomological collection sites through structured observations and indirect monitoring. These data are important because the proportion of bites received indoors during the hours when a person is potentially protected by a mosquito net can have profound impact on the effect size of the intervention and this can vary spatially [32]. In addition, the qualitative component will use in-depth interviews (IDIs), focus group discussions (FGD), and participant observations to better understand the reasons for behaviour patterns and key drivers of ITN use and non-use across the different types of ITNs [33] (Table 5). The gold standard to derive this kind of information would be to work with an anthropologist living within study sites, but cost limitations prohibited this. Sample sizes were designed to get a robust set of information from each district, across variations in key societal structures that could influence ITN behaviours. In all evaluation sites, the qualitative sampling frame should be understood as maximums potentially needed to achieve information saturation. There could possibly be fewer participants in each frame if saturation is achieved, or more could be requested if saturation is not achieved. Anthropological activities occur twice annually in Burkina Faso and Rwanda to correspond with expected periods high and low seasonal malaria transmission. They occur annually in Nigeria, and once in Mozambique due to budgetary constraints.

**Table 5:** Anthropological sample sizes for each activity by country

		Burkina Faso	Northern and Western Mozambique	Nigeria	Rwanda
In-depth interviews	Sample size	48 participants per district (144 total)		48 participants per LGA (192 total)	48 participants per district (144 total)
	Frequency	• 2019: July to September • 2020: March to May, July to September • 2021: March to May, July to September • 2022: March to May		• 2020: November to December • 2021: October • 2022: July to August	• 2020: July to August, November to December • 2021: March to April, July to August • 2022: March to April, July to August
Focus group discussions	Sample size	240 participants per district (720 total)	80 participants per site (480 total)	160 participants per LGA per year (640 total)	240 participants per district (720 total)
	Frequency	• 2019: July to September • 2020: March to May, July to September • 2021: March to May, July to September • 2022: March to May	• 2021: October to November	• 2020: November to December • 2021: October • 2022: July to August	• 2020: July to August, November to December • 2021: March to April, July to August • 2022: March to April, July to August
Structured observation	Sample size	70 households per district (210 total)		70 households per LGA (280 total)	
	Frequency	• 2019: July to September • 2020: March to May, July to September		• 2021: March to April • 2022: March to April	
Participant observation	Sample size	200 participants per district (600 total)		150 participants per LGA (600 total)	70 participants per district (210 total)
	Frequency	• 2019: July to September • 2020: March to May, July to September • 2021: March to May, July to September • 2022: March to May		• 2021: February to July • 2022: January to June	• 2020: November to December • 2021: March to April, July to August • 2022: March to April, July to August
Indirect monitoring	Sample size	70 participants per district (210 total)		90 participants per LGA (360 total)	200 participants per district (600 total)
	Frequency	• 2021: March to May, July to September • 2022: March to May		• 2021: March to April • 2022: March to April	• 2020: November to December • 2021: March to April, July to August • 2022: March to April, July to August
HLC-based observation	Sample size		9 households per district (54 total)		
	Frequency		Monthly starting October 2021		

*HLC* human landing collection, *LGA* local government area

#### In-depth interviews (IDI)

IDIs explore reasons for patterns of ITN use behaviour and are conducted after each observation period to gain an understanding of patterns observed. A maximum of two interviews per 24-h period with the same informant are conducted, for a maximum of 48 interviews per district or until saturation is reached (i.e., no new information is gained from subsequent interviews). Interviews are conducted using semi-structured guides and validation of observations. In Mozambique, questions regarding net use are incorporated into the durability monitoring assessments at baseline and again at 12 and 24 months, in lieu of IDIs.

#### Focus group discussions (FGDs)

After observations and IDI analysis has been completed, key themes are extracted, and FGDs are held with determined groups to explore factors in transmission risk, particularly those associated with ITN usage. Semi-structured guides that include validated themes that emerge through the analysis of observations and IDIs are used during each discussion.

#### Structured observation and indirect monitoring

These observations of human activity patterns are conducted in villages in each study district. This includes quantitative recordings of waking times, daily activities (including travel times), and time spent indoors and outdoors. Structured observational tools guide the data collection, and observations occur in the same villages as the entomological surveillance to correlate human and mosquito behaviour.

In Mozambique, approximately every 30 min during the HLCs, mosquito surveillance personnel record the proportion of people resting under an ITN indoors and outdoors (stratified by age) and the proportion of ITNs in use indoors and outdoors. In Burkina Faso, Rwanda, and Nigeria, indirect monitoring is used to gather data on time spent in and out of ITN protection. Participants use stopwatches for seven days to record the time they enter the protection of their net and the time they leave their net. All adults and up to two children (6 months of age or older) in each household participate in the collection of stopwatch data.

#### Participant observation

A trained observer participates in ongoing daily activities. Observations are conducted throughout the duration of the researcher’s stay in the village. These observations pertain to general malaria dynamics. ITN ownership and use are recorded in the observers’ logs on a regular basis, and de-identified notes from these logs are shared through weekly meetings to allow for refinement of structured observation and IDI instruments. In some areas, a direct observation approach is taken where research assistants visit their assigned village and conduct observations each day instead of living with host families and participating in daily life activities.

#### Selection criteria

The inclusion criteria for observations, IDIs, and FGDs include proximity to the study teams. Inclusion in participant observation is based on convenience sampling to ensure consistency and quality of the data over time. Individuals with cognitive impairment or underage individuals are excluded from the IDIs, FGDs, and participant observations. Heads of household unwilling and/or unable to give consent are excluded from structured observations.

### Durability monitoring

The retention time, physical integrity of mosquito nets and the length of time that the AI(s) remain effective on the net surface are critical to understanding the prolonged effectiveness of mosquito net interventions and may differ by location and/or product [34, 35]. As such, the durability of various net types will be monitored across each country. Methodologies for this component have been adapted from the PMI’s ITN Durability Monitoring Toolkit [36].

A three-stage sampling procedure is used, first to select fifteen clusters (villages) per location with probability proportionate to size, with the number of ITN microplanning data as the size variable. Next, within each selected cluster ten households are selected randomly from a list of community households to establish an ITN cohort for an annual follow-up. Within each household, all campaign nets are identified and labelled with a unique identifying number. Study nets distributed during each mass campaign are followed prospectively, with the baseline survey occurring within three to six months following the distribution campaign, and up to three annual surveys occurring thereafter.

#### Physical durability

During the baseline survey and subsequent follow-up surveys, the physical condition of each campaign net is assessed by examining the net and conducting a hole assessment to categorize the number of holes present and their size [37]. Repaired holes are documented but are not included in this assessment. Additional data are collected on household characteristics; number of ITNs received; exposure to messages on ITN care and repair; behaviour, attitudes, and perceptions; reasons for loss or absence of nets; and location, type of sleeping place, and washing and drying habits for campaign nets and for other ITNs not obtained from the campaign.

#### Insecticide durability

Critical to comparing the potential of the dual-AI ITNs is determining their ability to chemically impact (i.e., kill, repel, or reduce reproductive outputs) mosquitoes over time. During each follow-up at baseline, month 12, and month 24, a sample of 30 campaign ITNs, 2 from each of 15 clusters, from each district is selected for insecticidal effectiveness analysis. In households selected for bioassay testing, data are collected to confirm the selected ITN is from the campaign. In addition, information on the net’s use and washing patterns is collected. A replacement ITN of the same brand will be provided to households whose net is selected for appropriate bioassay testing, either by WHO cone tests [38] or another appropriate method [39]. Prior to initial distribution and at the last monitoring point (24 months), samples are also taken to perform chemical residue analysis.

The specific methodology for chemical residue testing and bioefficacy testing will be finalized in coordination with other partners, including IVCC, the London School of Hygiene & Tropical Medicine, PMI, the Liverpool School of Tropical Medicine, and the WHO. These methodologies will be published in separate protocols and are subject to modification as the understanding of best practices evolves. ITNs will be stored for potential reanalysis as these protocols evolve.

### Impact modelling

Given that the ecology of any setting is highly variable, clusters may not match initial conditions experienced by communities prior to the introduction of interventions. Unavoidable restrictions in the capacity of the pilots, and the logistical need to deliver nets on mass across large geographic settings for affordable campaigns, mean that not all covariates with potential to impact on malaria transmission can be monitored across all clusters (or districts). This may result in division of clusters being skewed in terms of the represented ecological situation at sites comparing one net class to another. Mechanistically, mosquito behaviours [32, 40], human activities [41], seasonal variables driving mosquito densities [42], baseline endemicity levels, and the level of pyrethroid resistance in the mosquito population [43, 44] can all alter the efficacy that would be measured or predicted for mosquito net interventions deployed in a given setting. The timing of the deployment of the intervention relative to seasonal peaks in malaria may additionally alter net impact [45].

Mathematical transmission models enable, while holding explicit assumptions, the exploration of potential intervention effects because they mechanistically capture how *Plasmodium falciparum* malaria is transmitted between human and mosquito populations. The falciparum malaria transmission model [46–49] that is employed for NNP has been described comprehensively elsewhere [42, 50] and the code is publicly available [51]. The epidemiological efficacy of mosquito nets [43] has been estimated through rigorous systematic reviews of bioassay data including susceptibility bioassays and experimental hut data [52]. These predictions have been validated by using the transmission model to predict the two cluster randomized trials (RCT) on PBO ITNs so far completed. (Although there is currently less certainty about the potential of IG2 or RG, a similar process can be carried out to validate prediction of these nets once RCTs are completed.)

The modelling team will use the pilot data for each country to specifically calibrate the transmission model parameters to reflect what can be understoodd about mosquito ecology, intervention use and human characteristics (e.g., treatment, other intervention use such as seasonal malaria chemoprevention, proportion of infectious bites received indoors or in bed) at the cluster or district level (depending on the granularity of the respective data sources). Predictions of efficacy can be made at the cluster (or district) level to help interpret what is observed during the trials and to explain the potentially distinct efficacy across regions receiving different classes of mosquito net.

### Cost-effectiveness of dual active ingredient insecticide-treated bed nets

There are two sources of incidence data that could be combined into the cost-effectiveness analysis. First, incidence rate ratios calculated from routine health management information system data can be used to provide summaries for each setting, allowing for the exploration of the range of cost-effectiveness potentially offered by the dual-AI ITNs. Second, individual-based stochastic models that capture the mechanisms of malaria transmission [47, 49] can be parameterized with the pilot study data including estimates for insecticide resistance (approximated as the proportion of mosquitoes surviving exposure to the discriminatory dose of pyrethroid at bioassay testing), treatment-seeking behaviour, timing of intervention deployment and subsequent adherence to net use by communities, mosquito species and related bionomics. The entomological efficacy of IG2, PBO, and standard pyrethroid-only ITNs have been separately determined using systematic review of experimental hut data [43, 52] (with that for RG ITNs to follow using additional bioassays and experimental huts allowing the inclusion of the impact of pyriproxyfen on oviposition and adult emergence rates [53]). These inform the transmission model to allow predictions to be made about epidemiological efficacy as resistance, approximated using the percentage survival of mosquitoes testing in the discriminatory bioassay, changes. The process is broadly able to predict pyrethroid- and PBO ITNs for the recent Tanzania trail [10, 44], and ongoing work will determine the validity of the framework systematically across multiple RCTs. Sensitivity analyses covering the range of parameter estimates observed during cross-sectional surveys can be performed for each cohort included in the pilot study using the transmission model framework. These models then provide a mechanism to predict the potential effectiveness in terms of cases averted or lives saved across other ecological settings.

The costs of each net distribution will be measured from a provider perspective and financial and economic costs calculated using an ingredients-based approach. Financial costs reflect the explicit expenditure of money associated with a programme, and economic costs reflect total resource use, or opportunity costs of a project, some of which may not be explicitly accounted for in a record of programme expenditures (such as volunteered time). The ingredients approach, or micro-costing, entails identifying all inputs to a process and assigning unit cost to each input. Costs are collected retrospectively for all campaign activities, including pre-distribution planning; household registration and community sensitization; net transportation and storage; distribution of nets to recipients; social and behaviour change communication; and monitoring and evaluation from financial and operational records, reports, pay scales, budgets, and invoices, as well as operational protocols and reports. Stakeholder interviews or resource-use survey instruments may be utilized to capture costs or resource inputs not reflected in financial or other records, such as volunteer time or assets used during the distribution but owned prior to the distribution (i.e., company vehicles), and national, regional, and local-level supervision time, or shared resources that are not included in standard financial reports. Additionally, primary cost data collection will be supplemented by a systematic review of published literature to ascertain uncertainty around measured cost estimates especially for use in probabilistic sensitivity analyses.

### Sampling considerations

#### Epidemiological power

The primary epidemiological outcome for which the evaluations are powered is malaria infection prevalence at the district level, as determined by RDTs in children aged 6 months to 5 years and in Rwanda all ages, assessed during annual community cross-sectional surveys. Within each study district, participants were targeted using a clustered sampling approach, clustering observations at a subdistrict level either by sector, health facility catchment, or village—whichever is most practical. Sample size calculations aim at 80% power to detect either a 30% (Burkina Faso and Rwanda) or a 15% (Mozambique and Nigeria) relative reduction in prevalence in an intervention district compared to a 5% reduction in prevalence observed in the standard pyrethroid-only ITN control district [10].

Sample sizes were estimated in PASS 15.0 (NCSS, LLC; Kaysville, Utah) using the following assumptions:

- Minimum of 15 households sampled per village.
- An average relative reduction in prevalence of 5% in the standard pyrethroid-only ITN study district.
- An average relative reduction in prevalence of 30% (Burkina Faso and Rwanda) or 15% (Mozambique and Nigeria) across the 2-year follow-up.
- Higher than normal estimates of within-cluster standard deviation (0.3) and within-pair variability (CVM = 0.66) to accommodate the likelihood of higher-than-normal homogeneity within non-randomized ‘clusters’.
- A one-way test with Power = 0.80 and Alpha = 0.0125 (lower than normal to accommodate Bonferroni’s correction for multiple comparisons of the same control district to the different intervention districts).

#### Entomological sampling

Mosquito sampling approaches for these observational studies were designed to maximize available vector surveillance resources, and while a priori statistical criteria were not considered, post hoc descriptions of sampling variability and resulting statistical power appropriate to the tests used will be performed.

#### Anthropological sampling

For structured observations, a paired sampling design, whereby villages are chosen to correspond to where entomological surveillance is also happening, is applied. Informants are chosen based on specific activities identified during direct observations. A list of activity groupings is developed to ensure consistency and modified as necessary based on new information from the direct observations. Purposive selection is used to identify informants for interviews, based on their participation in target groups. The number of focus groups is determined from the salient categories; each focus group includes between six and ten participants identified during interactions and conversations with research assistants. Participant observation uses a convenience sampling methodology to observe key activities related to malaria transmission.

#### Sample size in durability monitoring surveys

Sample sizes for the monitoring of ITN physical durability are based on recommendations from [36] and are powered to detect a 9%-to-10%-point difference between locations (products) if the assumed median survival is three years. This represents approximately a 0.5 median survival difference that can be detected as statistically significant [36]. Sample sizes for monitoring ITN insecticidal durability follow WHO recommendation for Phase 3 testing of long-lasting insecticidal nets [38].

### Ethics approval and consent to participate

The current study involves human subjects (epidemiological and anthropological investigations) and nonhuman subjects research (entomological surveillance) in rural communities, as well as secondary use of routine health data. To ensure the safety and well-being of all participants involved in this research, seven institutional review boards reviewed country-specific protocols. In addition, annual reviews will be conducted for the duration of the project to ensure compliance with the methodologies outlined in the protocol.

Prior to entering communities, meetings are organized with the village chiefs, their councils and other relevant authorities, together with the heads of households. Researchers introduce the project and themselves, provide information on who will be conducting which activities, and obtain local authorization.

In all data collection procedures, the participants are informed of the voluntary nature of their participation; of the right to avoid any question with which they may feel uncomfortable; and of their right to withdraw from the study at any time without having to provide a reason and with no consequences for them or their families. Having received an ITN through mass distribution is not a prerequisite for participation.

For the cross-sectional survey, individual informed consent is obtained by trained study personnel from the caregivers of children aged 6 to 59 months at the time of the survey. In Rwanda, consent is obtained from parents/guardians of children aged 6 months to 17 years, assent is obtained from participants aged 7 to 17 years, and consent is obtained from participants aged 18 and older. Individual informed consent is also sought from participants in IDIs, FGDs, and indirect monitoring related to the anthropological component. Consent is sought from the heads of households for structured observations.

The entomological surveillance at homesteads in sentinel villages, though qualified as nonhuman subjects research, requires local leaders and household permission, usually from the head, prior to the performance of any procedure. The granted permission may, for any reason, be withdrawn at any time.

Due to the invasive nature of the observational procedures (access to/inspection of dwellings), especially in anthropological investigations, an initial community sensitization takes place. Any concerns that are brought up during the initial meeting and throughout the study are elevated to the principal investigators to determine if changes need to be made to study procedures. Participant observation is conducted in settings of public activities and does not usually require individual informed consent; however, in the current study, permission is obtained from village chiefs presiding over the public spaces.

### Risks and unexpected adverse events

There is minimal physical and psychological risk associated with this observational study. However, the study team works with the NMCPs and ITN distribution partners to ensure that any potential adverse events related to ITN use are reported appropriately and refers participant concerns to the nearest appropriate health facility.

While severe malaria is not diagnosed as part of study activities, any suspected cases of severe malaria are to be referred immediately to the nearest appropriate health facility. If a household or family member, present at the time of the visit, complains of fever, an RDT will be offered and, if positive, the household member will be offered malaria treatment. If negative, they will be asked to seek medical care as normal. This is not considered part of the research but is done as a broader community benefit. Any unanticipated problem that poses a risk of harm to study participants is reported to all ethics committees within the specified time frame.

The volunteers participating in mosquito collections are screened for malaria infection every two weeks using RDTs and treated with antimalarials if they test positive.

### Analysis and data management

#### Data storage

All data will be de-identified prior to analysis to protect participant confidentiality. Households and participants in cross-sectional and entomological surveys will be assigned unique identification codes. For anthropological research, personal information will be replaced with unique codes assigned to individuals interviewed. The data collected are protected in secure file cabinets or password-protected computers and will be backed up on servers. Electronic data collected on password-protected tablets is transferred, daily, to local servers. Data collection devices are handled only by study staff and kept in a secure location.

#### Epidemiological surveillance analysis

The trials are planned under a superiority, difference-in-differences (DiD) framework. The comparisons will consist of one-sided tests of the null hypothesis that the outcomes (reductions in parasite prevalence or case incidence rate from baseline to end line) in each intervention district are statistically the same as the outcomes in the appropriate control district control arm.

Malaria infection prevalence, determined by RDT tests during the cross-sectional surveys, will be analysed by an appropriate difference-in-differences model comparing changes in prevalence from year to year across study districts (intervention type). Considering sample size constraints, a single indicator age group will be targeted using a clustered sampling approach, clustering observations at a sub-district level (either by sector, health facility catchment, or village—whichever is most practical). Statistical analysis is done using Stata (version 14.2 or later). A difference-in-differences approach will assess matched-pair comparisons of the mean change in malaria prevalence from year to year, before and after ITN distribution across each study districts (intervention/control) comparison. The approach taken within each evaluation setting is powered independently to compare changes in prevalence observed in the ITN intervention districts to changes in the prevalence observed in the appropriate ITN control district. However, given the proposed study design and sampling frame, secondary post-hoc analyses, with Bonferroni’s correction for multiple comparisons, will likely be able to detect an effect size difference of around 20% between any two ITN types at 24 months. The protective efficacy of dual-AI ITNs is determined by multilevel logistic regression model for each year, adjusted for age and individual ITN use the previous night, with clustering at the district level.

For passive case detection (PCD), an observational time-series study design using malaria surveillance data from the district health system will be used to compare trends in malaria case incidence in all age groups using a difference in differences approach. Monthly crude case incidence rates will be calculated at the health facility level by dividing the number of all positive malaria tests (RDT and blood smear) obtained that month by the estimate health facility catchment area population. Appropriate multivariate regression models accounting for the clustering of health facilities by district and for possible confounding factors (e.g., a negative binomial regression analysis of monthly case incidence rates likely to include rainfall amount and health facility catchment area population size), will also be utilized to estimate incidence rate ratios and incremental protective efficacy for IG2, PBO and RG ITNs. Alternatively, test positivity rates (the number of negative and positive test results obtained from suspected cases at the health facility level, stratified by study arm) may also be used to obtain odds ratio estimates from an appropriate logistic regression model. Raw datasets will be cleaned and transformed using Microsoft Excel (Microsoft Corp., Redmond WA) and Tableau v10 (Tableau Software Inc., Seattle WA) and all formal statistical analyses will be done using Stata v14 (StataCorp LLC, College Station TX).

ITN ownership, use, and coverage estimates will be calculated using standard methods recommended by the Surveillance, Monitoring and Evaluation Working Group of the RBM Partnership to end Malaria (https://endmalaria.org/node/990/related-material).

#### Entomological surveillance analysis

Vector species–specific entomological data are analysed by comparing changes in monthly vector density trends across districts using a zero-adjusted negative binomial regression model. Species-specific biting behaviours, entomological inoculation rates, and insecticide resistance patterns are compared again using appropriate t-tests or similar methods.

#### Anthropological analysis

Data on human behaviour are analysed using a mixed methods approach, with a quantitative component nested within a broader qualitative component. The quantitative data measure the actual time individuals spend unprotected by ITNs, including classifications of types of activities and locations. This information will aid in the modeling and cross-analysis with entomological and epidemiological data. The qualitative component investigates the human behaviours that influence patterns of exposure, providing a crucial framework of analysis for the quantitative data, and are coded and analysed using ATLAS.ti or NVivo software to identify key themes.

#### Durability monitoring data analysis

The analysis of the durability monitoring component follows a prospective cohort study design of cohort nets distributed through a mass campaign, in-line with WHO and PMI standards [36, 37] for estimating the mean useful life of an ITN in operational settings. Nets are assessed at baseline and again at 12 and 24 months. The measurement for month 36 will not occur in Mozambique or Nigeria due to the time frame limitations of the grant.

Attrition, survivorship, and insecticidal bioefficacy and decay are determined at all time periods and are analysed for differences by each ITN type. Protocols for measuring the insecticidal effectiveness and insecticide content of non-pyrethroid ITNs are being developed and agreed upon throughout the partnership and will be published separately. The ITN samples are stored if reanalysis is needed once protocols have been standardized.

#### Costing and cost-effectiveness data analysis

The time horizon for cost analysis fits the duration of study, respective to setting. Costs are presented as a table of inputs, unit prices, quantities, and line item and activity codes, as possible. Costs are divided into capital and recurrent costs, based on the lifetime of the goods or service being purchased. Capital costs are discounted in the economic analysis using lifetimes determined through stakeholder interviews, expert information, and prior published literature and a discount rate of 3% (in base case scenario analysis). Varying discount rates and lifetimes are examined in sensitivity analyses. Both financial and economic analyses will be conducted. These two types of analysis show (1) financial costs (what the explicit expenses of running a programme are) and (2) economic costs (the value of all resources used during study duration). In the financial analysis, capital costs are not discounted and are applied in full at the time of purchase. All costs must be converted to a common year and currency, to ensure comparability between settings with different inflation rates and currency values. Costs are converted from local currency to US dollars using the exchange rate for the year the cost analyses are conducted. The Consumer Price Index adjustment is used to inflate or deflate all costs. Next, costs are split into two categories: tradable costs (e.g., nets) and non-tradable costs (e.g., personnel, training, supervision, social and behaviour change communication). Non-tradable costs are further converted to 2020 international dollars. This is accomplished using the purchasing power parity ratios for the countries in which the costs were incurred. For comparability and interpretability across settings, results for all countries will be presented in both 2020 US dollars and international dollars.

Costs are reported in three ways: total economic cost of the programme, total cost by activity and line item, and the total cost per output. Total cost is simply total economic cost of the mass distribution of long-lasting insecticide-treated nets over their expected lifetime. The total cost is then broken down by activity and line item. Finally, the total cost is reported as a ratio over selected outputs, such as nets distributed, sleeping spaces covered, person-years of protection, and treated net years.

## Discussion

Recent gains in global reduction of malaria burden are threatened by increases in vector resistance to insecticides used in ITNs and IRS. Dual-AI ITNs provide an opportunity to accelerate progress towards reducing malaria morbidity, especially in high-risk populations with pyrethroid-resistant vectors. Dual-AI ITNs have been shown to have a higher killing impact on pyrethroid-resistant mosquitoes in experimental huts, which, if validated in the field, could further decrease transmission [13, 54, 55]. However, there remains limited programmatic evidence on the epidemiological efficacy and effectiveness—and durability of the dual-AI IG2 and RG ITNs. There are ongoing cluster randomized controlled trials occurring in coordination with NNP [56], and these are the gold standard method to empirically quantify the efficacy of these mosquito net interventions [57]. However, effectiveness determined by operational pilots in which explanatory information is collected in a robust way serve critical and complementary purposes. First, evaluations of full-scale implementation allow for measurement in a variety of settings, reflecting the diversity in the ecology and social patterns of the pilot locations, which is broadly important to the global malaria control community. Impacts from vector control can be quite different depending on the setting [10, 58, 59]. While randomized control trials are not possible in every location, pilot studies provide expanded data to further understand how these dual-AI ITNs will likely perform during mass scale-up efforts and help identify what other factors are important in considering scale-up and potential impact elsewhere. This is increasingly important within the context of scale-up of other malaria interventions, especially as the costs of ITNs rise. Second, by design cluster randomized control trials track cases in a robust and controlled manner that is infeasible in more routine health care settings given the increased levels of staff required to conduct the trials, and therefore do not reflect the experiences of NMCPs. To make the findings applicable to broader country contexts, these pilots provide analysis of routine data and are reflective of the impacts that may be seen by NMCPs during scale-up. A focus on the utility and breadth of information collected for each component will enable NMCPs and funding agencies to determine the most useful data with which to make decisions about the optimal placement of these nets based on burden, vector bionomics, human acceptability, quality, and efficiency.

The epidemiological data from routine health facilities and annual cross-sectional surveys from the four countries with varying malaria endemicity will be key in providing supporting evidence for broader scale-up and policy. In addition, the anthropological data will enhance understanding of community perceptions of the dual AI ITNs that impact their uptake and use and therefore their effectiveness in vector control. In understanding the reasons for any differences in cost-effectiveness, it is critical to understand ITN impacts on the primary and secondary vector species. The results from the entomological surveillance will also provide key evidence on the temporal and spatial patterns of vector species distributions, entomological inoculation rates, and mosquito mortality in the short to medium terms for IG2 and RG, as compared to pyrethroid ITNs or other PBO ITNs in settings where insecticide resistance is high. Evidence from physical and insecticidal durability monitoring will provide NMCPs with key information on the performance and estimated useful life of the different ITN types under different conditions and across countries, which will in turn influence their cost-effectiveness and timing of replacement net distribution. Finally, data from each component of the study will be available to inform mathematical modeling work designed to help describe and predict the effectiveness and cost-effectiveness of different net types in various malaria transmission settings and intervention contexts across sub-Saharan Africa.

One of the strengths of the NNP pilot evaluations is that they take advantage of natural comparisons that arose as the result of normal operational decisions made by partner national malaria programmes. This allows the impact of various ITN deployment strategies (including a formal evaluation of PBO ITNs in West African contexts with high intensity pyrethroid resistance caused by multiple mechanisms) to be evaluated rapidly and in real-world implementation settings, using programmatic data and public health outcomes that are most useful for national stakeholders, both for monitoring progress toward national goals and for informing future vector control decisions. However, the eco-observational, pilot designs also have many important limitations. First, though district-level observations are matched in time and, to the greatest extent possible, in ecology and geography as well, there are challenges in generating supportive datasets needed to account for fundamental differences that may exist between study districts and for potential confounding and/or co-variable factors.

This was particularly true given that campaign ITN procurement and distribution logistics required the stratification and delivery of different ITN types at the district or provincial level, so that all districts—the unit of analysis used here—within a single province typically received the same ITN type. As such, it was necessary to limit these pilot evaluations to contiguous and/or nearby districts from neighboring provinces. This approach to selecting study districts means that baseline comparisons of malaria burden and vector ecology and bionomics varied substantially both within and across the different evaluation settings, capturing real-world diversity in transmission settings but also complicating the interpretation of the observational results. While this does limit their broad generalizability, it is reflective of the diversity in which these products will be taken to scale and will provide insights into the diversity of effectiveness likely encountered through massive scale-up.

Routine health facility data provide real time insight to malaria cases and trends, but are often limited by poor data integrity, limited capacity for quality control, and supply shortages, leading to underestimation of true malaria case incidence rates. However, this robust multi-country protocol aims to ensure that the breadth of information and multiple methods of evaluation will strengthen conclusions on the cost-effectiveness of these interventions across a variety of settings in a way that a single evaluation or single measure could not.

Another source of variability, and a limitation of the study design particularly important when considering preliminary and interim results, is the relatively brief periods that were available for baseline, pre-intervention data collection given the need to align study activities with already existing regional and national-level universal coverage campaigns. The timing of campaign activities differed across study districts and so does the resulting length of time that each intervention has been deployed to date.

Due to the complexity of national malaria programming and each net distribution campaign, the protocols are not identical; however, the key ITN indicators have been harmonized across studies. A single data analysis framework has been codeveloped by study partners and is supplemented by local nuance for each individual analysis, including possibilities such as duration of net ownership, differences in measured baseline characteristics, and data gaps. In each case, if there is deviation from the main analysis plan, these actions will be described.

It is also worth noting these NNP pilot studies are not able to estimate the general impact of any (or all) ITNs relative to no ITNs, and instead they are attempting to quantify the incremental impact of dual-AI ITN types relative to standard, pyrethroid-only ITNs that still provide protection from malaria—particularly when access and use are high.

Finally, all these operational challenges were exacerbated by the ongoing COVID-19 pandemic response, and steps to account for these changes will be contextualized for during any final analysis.

## Conclusion

These pilot evaluations, across 16 varied districts in four countries, in four broad regions of sub-Saharan Africa, will provide important and comprehensive evidence on the effectiveness and cost-effectiveness of dual-AI ITNs that will support NMCPs, donors, implementing agencies, policymakers, and other national and regional stakeholders in their decision-making and planning for malaria control activities using these promising new tools.

## Supplementary Information

**Additional file 1. Table S1.** Summary of insecticide-treated bed net products.

**Additional file 2. Annex S1.** Country-specific context.

## Data Availability

The datasets used and/or analysed during the current study are available from the corresponding author upon reasonable request.

## Abbreviations

CDC: US Centers for Disease Control and Prevention
CDCLT: US Centers for Disease Control and Prevention light trap
COVID-19: Coronavirus disease 2019
dual-AI: Dual active ingredient
FGD: Focus group discussion
Global Fund: The Global Fund to Fight AIDS, Tuberculosis and Malaria
HLC: Human landing collection
IDI: In-depth interview
IG2: Interceptor® G2
IRS: Indoor residual spraying
ITN: Insecticide-treated bed net
*kdr*: Knockdown resistance
LGA: Local government area
MOPDD: Malaria and Other Parasitic Diseases Division
NMCP: National Malaria Control Programme
NNP: New Nets Project
PBO: Piperonyl butoxide
PMI: US President’s Malaria Initiative
RBC: Rwanda Biomedical Centre
RDT: Rapid diagnostic test
RG: Royal Guard®
WHO: World Health Organization
