# Supplementary material for: Design and methods for a quasi-experimental pilot study to evaluate the impact of dual active ingredient insecticide-treated nets on malaria burden in five regions in sub-Saharan Africa
Source: Malar J. 2022 Jan 10;21:19. doi: 10.1186/s12936-021-04026-0 (PMC8744060; doi:10.1186/s12936-021-04026-0)
Supplement: Supplementary file 1 — Additional file 1. Table S1. Summary of insecticide-treated bed net products. [file 12936_2021_4026_MOESM1_ESM.docx]

# Additional file 1. Table S1.

ITN products and relevant current recommendation statuses for ITNs that may be used in mass and continuous distributions. Designation specifies the category of each ITN, based on WHO’s lists of prequalified vector control products [60].

**Table S1 Summary of insecticide-treated bed net products**

| **Product name** | **Abbreviation** | **Manufacturer** | **Product type** | **WHO policy recommendation** | **Designation in protocol** |
| --- | --- | --- | --- | --- | --- |
| Olyset^®^ Duo | OD | Sumitomo Chemical Co., Ltd. | Pyriproxyfen + permethrin incorporated into polyethylene | No specific recommendation; data from epidemiological trials awaited | Dual-AI ITN |
| Olyset^®^ Plus | OP | Sumitomo Chemical Co., Ltd. | Permethrin + PBO incorporated into polyethylene, all panels | Conditional recommendation^[[1]](#footnote-1)^ [61] | PBO ITN |
| PermaNet^®^ 3.0 | PN | Vestergaard Frandsen Holding SA | Combination of deltamethrin coated on polyester with strengthened border (side panels) and deltamethrin + PBO incorporated into polyethylene (roof) | Conditional recommendation^d^ [61] | PBO ITN |
| Veeralin^®^ | VL | V.K.A. Polymers Pvt. Ltd. | Alpha-cypermethrin + PBO incorporated into polyethylene, all panels | Conditional recommendation^d^ [61] | PBO ITN |
|  |  |  |  |  |  |
|  |  |  |  |  |  |
| Interceptor^®^ G2 | IG2 | BASF SE | Alpha-cypermethrin and chlorfenapyr coated on polyester | No specific recommendation; data from epidemiological trials awaited | Dual-AI ITN |
| Royal Guard^®^ | RG | Disease Control Technologies, LLC | Alpha-cypermethrin and pyriproxyfen incorporated into polyethylene, all panels | No specific recommendation; data from epidemiological trials awaited | Dual-AI ITN |
| Standard nets | SN | Various | Various pyrethroids either coated onto polyester or incorporated into polyethylene | Recommended since 2001 | standard pyrethroid-only ITNs |

Abbreviations: dual-AI, dual active ingredient; ITN, insecticide-treated bed net; PBO, piperonyl butoxide; WHO, World Health Organization.

# References

- - 1. WHO. Prequalification vector control. Geneva, World Health Organization. <https://www.who.int/pq-vector-control/prequalified-lists/en/>. Accessed 18 Feb 2021.
    2. WHO. Global Malaria Programme: Conditions for deployment of mosquito nets treated with a pyrethroid and piperonyl butoxide. Geneva, World Health Organization. [https://apps.who.int/iris/bitstream/handle/10665/258939/WHO-HTM-GMP-2017.17-eng.pdf. Accessed 11 May 2021](https://apps.who.int/iris/bitstream/handle/10665/258939/WHO-HTM-GMP-2017.17-eng.pdf.%20Accessed%2011%20May%202021).

1. A conditional recommendation indicates that “benefits outweigh harms for the majority, but not for everyone. The majority of patients would likely want this option.” A conditional recommendation indicates that the quality of evidence is low. [3] (WHO guidelines for Malaria v1.3. https://app.magicapp.org/#/guideline/4870. Accessed March 12 2021). [↑](#footnote-ref-1)
