# Supplementary material for: Design and methods for a quasi-experimental pilot study to evaluate the impact of dual active ingredient insecticide-treated nets on malaria burden in five regions in sub-Saharan Africa
Source: Malar J. 2022 Jan 10;21:19. doi: 10.1186/s12936-021-04026-0 (PMC8744060; doi:10.1186/s12936-021-04026-0)
Supplement: Supplementary file 2 — Additional file 2. Annex S1. Country-specific context. [file 12936_2021_4026_MOESM2_ESM.docx]

# Additional file 2. Country-specific context.

## Burkina Faso

### General background

Burkina Faso is a 274,200 km^2^ West African Sahelian landlocked country located at a transitional zone between the arid Sahara in the north and the Sudanian zone in the south [62]. The start, duration, and total number of rainy days is therefore highly variable in space and time and defines three ecoclimatic zones: Sahelian zone in the north, Sudanian zone in the south, and Sudano-Sahelian zone in between, with a total annual rainfall and average annual temperature of less than 600 mm/29°C, 900–1,200 mm/28°C, and 600–900 mm/27°C, respectively [63]. Nearly 80% of the country’s population work in the agriculture sector and around 70% reside in rural areas [62].

### Local malaria control context

Malaria occurs throughout the year in Burkina Faso, with a peak during the rains between June and October. The 2014 Malaria Indicator Survey estimated malaria prevalence at 45.7% [64]. Several control interventions have been scaled up in a relatively short time in Burkina Faso. The use of artemisinin-based combination therapies, namely artesunate-amodiaquine and artemether-lumefantrine, for uncomplicated malaria was adopted in 2005, and these therapies became available at health facilities in 2007 [65]. Artesunate for severe malaria was adopted in 2012 and made available in 2014 in severe malaria treatment kits at health facilities [21, 66]. Malaria home management by community health workers was pilot tested in 2008 and rolled out countrywide in 2010 [67]. From 2010 to 2013, indoor residual spraying (IRS) was implemented in one health district, Diébougou, and the intervention was halted in 2013. IRS implementation resumed in 2017 in three districts: Kampti, Koungoussi, and Solenzo [68]. Countrywide, insecticide-treated bednet (ITN) mass distribution campaigns were conducted in 2010, 2013, and 2016, with administrative coverage rates of 95.6%, 96%, and 97.41%, respectively [69, 70]. Additionally, population coverage achieved through the mass distribution campaigns has been supplemented by regular distribution of ITNs at all public health facilities through routine antenatal and expanded immunization programs. Malaria Indicator Surveys, however, showed ITN ownership rates of 90% and 75% in 2014 and 2018, respectively. In 2018, only 33% of households had at least one ITN for two members and only 44% were using their bednets [71]. Seasonal malaria chemoprevention with sulfadoxine-pyrimethamine plus amodiaquine in the high malaria transmission season has superseded intermittent preventive treatment in children in which the same drug was administered to children on a schedule matching that of the expanded program on immunization. Lastly, to increase health care seeking at public health facilities, a free of charge health care policy for children under 5 years was implemented in 2016.

### Study sites

Cascades and Boucle du Mouhoun Regions have been prioritized for the introduction of Interceptor^®^ G2 (IG2; BASF SE) based on the high prevalence of malaria [72] and a well-documented and characterized history of resistance to multiple pyrethroids (including permethrin and deltamethrin) by multiple mechanisms in local *Anopheles gambiae* s.l. populations (*kdr* target-site mutations as well as metabolic resistance involving oxidase enzymes) [9, 30, 73]. However, due to a limited number of IG2 ITNs, the totality of health districts in the southwestern region could not be covered.

A subset of five health districts, Tougan, Nouna, Banfora, Gaoua, and Orodara, will be included in the study (shown on the map in Figure 1). These districts had a combined population size of nearly 1.6 million people and 231 health facilities as of 2017. The overall malaria incidence per 1,000 people in 2017 was 535 in Nouna, 722 in Gaoua, 370 in Tougan, 729 in Banfora, and 631 in Orodara. The Nouna, Gaoua, Tougan, Banfora, and Orodara health districts are served by 51, 9, 41, 46, and 39 primary health facilities, respectively. They have similar malaria transmission dynamics and consistencies in other malaria control interventions. In addition, their baseline characteristics, determined through routine data provided by in-country stakeholders, were comparable across the districts in underlying malaria prevalence, incidence, vector species composition, and insecticide resistance status, and their climate and geographies are similar.

IG2 ITNs will be distributed in Tougan and Banfora; in the two comparator districts, Nouna and Gaoua, standard pyrethroid-only ITNs will be distributed. Orodara will receive piperonyl butoxide (PBO) ITNs, providing additional important context to support the decision-making process in Burkina Faso. Within each district, ITNs routinely distributed at health facilities will be of the same type as those distributed during mass campaigns.

## Mozambique

### General background

Mozambique is located on the coast of southeastern Africa between South Africa and Tanzania. The country is sparsely populated by 28 million people, and only 36% live in urban areas, including 1 million in the capital, Maputo [74, 75]. There is considerable linguistic diversity in Mozambique: Portuguese is the official language and 26.1% of Mozambicans speak Macua; 8.6% speak Changana; and the rest speak other local languages, of which Lomwe, Sena, and Makhuwa are common in the study districts. The religious makeup of the country is 59.8% Christian, 18.9% Muslim, 4.8% other, and the remaining 16.5% reported either having no religion or did not specify [76]. The communities that make up the study districts are mostly rural, and the chief economic activity is smallholder agriculture, primarily the cultivation of rice, maize, and cassava [76]. In 2017 the literacy rate was estimated to be 60.1% nationally (72.6% for males and 50.3% for females) [77].

### Local malaria control context

Malaria is endemic throughout Mozambique. The country experiences year-round transmission, and risk is heightened during the rainy season, typically from December to April. Malaria cases account for 42% of deaths in children under 5 years and 29% of all deaths overall, yet there are large differences in malaria prevalence and transmission by region. The 2018 combined Survey of Indicators on Immunization, Malaria, and HIV/AIDS showed that prevalence in children under 5 varied throughout the country, ranging from 1% in Maputo Province in the south to 57% in Cabo Delgado Province in the north. Prevalence was generally higher in the northern region (44% to 57%) than in the southern region (1% to 35%), and prevalence in rural areas was more than double that of urban areas (47% compared to 18%). Two provinces, Zambezia and Nampula, represented almost 40% of the national malaria burden. The 2018 indicator survey also showed improvement in ITN coverage compared to the 2011 Demographic and Health Survey: the number of households with at least one ITN increased from 51% to 82%, as did the proportion of children under 5 years and pregnant women reporting having slept under an ITN the night before the survey (36% to 73% and 34% to 77%). However, the number of malaria cases reported increased dramatically from 2012 to 2017, from 3.1 million to more than 8.9 million cases each year, which may be due in part to improved reporting through the routine health management information system [74].

### Study sites

The national malaria control program prioritized the targeting of dual active ingredient ITNs to two provinces, Manica and Niassa, based on moderate to high malaria infection prevalence rates observed reported in the 2018 combined indicators survey, documented pyrethroid resistance in local vector populations, pre-planned IRS operations targeting the highest-burden districts of Zambezia Province, and the time between net availability and campaign plans. In addition, PBO ITNs were targeted to Cabo Delgado and Tete Provinces based on insecticide resistance patterns. The timing of the planned PBO campaign in Cabo Delgado (July 2019) excluded this province from this study.

Six districts, covering two separate study areas (referred to here as the West and North evaluations) in Niassa and Zambezia Provinces in the north and Manica, Sofala, and Tete Provinces in the west were selected for enhanced study activities to help measure the impact of IG2, Royal Guard^®^ (RG; Disease Control Technologies, LLC), and PBO nets on malaria transmission (see the map in Figure 2). In general, Mozambique makes ITN distribution decisions at the provincial level. An exception was made for Niassa due to the inability to secure enough RG nets to cover the whole province. This provides an opportunity to conduct an observational comparison of RG and IG2 ITNs in Niassa with standard ITNs that are being distributed in Zambezia in the North evaluation. In addition, IG2 nets will be distributed throughout Manica Province, Sofala Province will receive standard ITNs, and Tete Province will receive PBO ITNs. This will allow for an observational comparison between the three ITN types and standard ITNs in the West evaluation. While choices for comparison are limited to districts within a province receiving a single net type, we have made every effort to choose neighboring study districts sharing a common ecology and socioeconomic status. Comparator districts are similar with respect to vector species composition and known insecticide resistance profiles, and general malaria burden (prevalence and estimated case incidence).

## Nigeria

### General background

Nigeria is in West Africa, bordered by Niger to the north and Benin and Cameroon to the west and east, respectively. Nigeria is the most populous country in Africa: an estimated 211 million people [78]. The country is divided into 36 states, which are further subdivided into 774 local government areas (LGAs).

### Local malaria control context

Malaria is endemic in Nigeria and a major public health concern, especially for children under 5 and pregnant women. In 2017, there were an estimated 53.7 million malaria cases in the country, representing almost 25% of the entire global burden [79]. Seventy-six percent of the population lives in areas defined by the World Health Organization as at high risk of malaria transmission, while the remaining 24% lives in areas of moderate to low transmission [80].

The primary malaria prevention strategy in Nigeria is the universal distribution of ITNs. The 2018 Demographic and Health Survey indicated that 62% of households owned at least one ITN, and 43% of those surveyed reported having slept under an ITN the night before [81]. In addition, the National Malaria Strategic Plan 2014–2020 includes targeted scale-up of IRS and expanded larval source management as part of an integrated vector management strategy, though IRS is not yet widely implemented [80]. The dominant malaria vector species group in Nigeria (*Anopheles gambiae* s.l.) has been shown to bite readily both indoors and outdoors and has demonstrated moderate to high levels of resistance to pyrethroids, the class of insecticide used on ITNs currently distributed throughout the country.

### Study sites

The four study LGAs are Asa and Moro in Kwara State and Ife North and Ejigbo in Osun State (shown on the map in Figure 3). The population in these areas ranges from 100,000 to 150,000 people. Study LGAs are in the rain forest or savannah ecological zones and are peri-urban and agricultural. The dominant languages are Yoruba and English, and the most common religions practiced are Islam, Christianity, and traditional religions. Though malaria transmission occurs year-round in Kwara and Osun States, there are seasonal peaks in case incidence and mosquito abundance, typically highest between September and November, during the rainy season. Study activities will be conducted in parallel in the pilot study LGAs, and the impacts of the distribution campaign will be compared across net types. Especially appealing about the proposed study sites is that the US President’s Malaria Initiative and national malaria control program recently documented and characterized widespread, high-intensity resistance to multiple pyrethroids (including permethrin, deltamethrin, alpha-cypermethrin, and lamdacyhalothrin) by multiple mechanisms (including metabolic resistance partially mitigated by PBO), highlighting the opportunity to evaluate this dual active ingredient ITN product in the type of environment where it is most needed*.*

## Rwanda

### General background

Rwanda is a landlocked country in Central Africa bordered by Burundi, Democratic Republic of the Congo, Tanzania, and Uganda. With 12 million people living within its 26,000 square kilometers, the country is one of the most densely populated in Africa [82]. Over 80% of the population resides in rural areas [83]. The country’s growing service sector provides over 50% of GDP, while the agricultural sector contributes roughly 30%. [82, 84]. Despite a slow annual population growth rate of 1.2% experienced in the 90s, between 2002 and 2012, the population grew by an average of 2.6% per year [83]. Recently the fertility rate has declined, going from 6.1 in 2005 to 4.2 in 2014 [85]. Kinyarwanda is the primary language, followed by English and French. Kiswahili is also spoken in select areas bordering countries where it is widely spoken. The religious background of the country is 93% Christian, 2% Muslim, and less than .5% reporting no religious affiliation [84]. To foster a unified identity and to continue reconciliation efforts after the 1994 genocide, the government introduced a new flag and national anthem in 2001 [86].

### Local malaria control context

Malaria transmission is high throughout the year, but peaks from April to June and from October to December following the two rainy seasons. Approximately 7% of children under 5 and 11% of children 5 to 14 tested positive for malaria by microscopy, according to the 2017 Malaria Indicator Survey. In both cases, prevalence among children in rural areas and children in the lowest wealth quintile was higher than among those in urban areas and those in the highest wealth quintile [20]. From 2005 to 2011, Rwanda significantly reduced its malaria burden, with overall incidence declining 85%, through implementation and scale-up of interventions. From 2012 to 2016, however, malaria incidence increased each year. The largest increases were observed in districts in Southern and Eastern Provinces. The Rwanda Biomedical Centre’s Malaria and Other Parasitic Diseases Division attributed this increase to several factors, including low universal ITN coverage, vector resistance to pyrethroid insecticides, and improvements in health facility reporting and availability of rapid diagnostic tests and artemisinin-based combination therapies. In late 2016 and early 2017, the government of Rwanda distributed more than 5 million ITNs through a mass distribution campaign and implemented IRS with an organophosphate insecticide, expanding coverage from three to five districts. From 2016 to 2017, national incidence stabilized [87]. According to the 2017 Malaria Indicator Survey, 84% of households reported owning at least one ITN, 92% of which were obtained from mass distribution campaigns, 4% from immunization visits, and 2% during antenatal care visits. Sixty-four percent of household populations reported sleeping under an ITN the night before the survey was conducted, including 69% of pregnant women and 68% of children under 5 years [87].

### Study sites

Three districts will serve as primary study sites for the pilot evaluations: Nyamagabe will receive standard ITNs, Karongi will receive IG2 ITNs, and Ruhango will receive standard ITNs and IRS. Ruhango and Nyamagabe are in Southern Province, and Karongi is in Western Province (see the map in Figure 4). Baseline characteristics show comparability across the districts in underlying vector species composition and insecticide resistance status, as well as general climate and geographic similarities. Within each district, ITNs routinely distributed at health facilities will be of the same type as those distributed during the mass campaign.

## References

- - 1. Central Intelligence Agency The World Factbook: Burkina Faso. <https://www.cia.gov/the-world-factbook/countries/burkina-faso/>. Accessed 2 Jul 2021.
    2. World Bank Group: Climate Change Knowledge Portal, Burkina Faso. <https://climateknowledgeportal.worldbank.org/country/burkina-faso/climate-data-historical>. Accessed 2 Jul 2021.
    3. Institut National de la Statistique et de la Démographie, Programme National de Lutte contre le Paludisme (PNLP), ICF International. Enquête sur les indicateurs du paludisme au Burkina Faso . Ouagadougou, Burkina Faso: PNLP; 2015.
    4. Ministère de la Santé. Plan stratégique 2011-2015 de lutte contre le paludisme au Burkina Faso. Ouagadougou, Burkina Faso: Ministère de la Santé; 2011.
    5. US Agency for International Development (USAID). Plan opérationnel de l’USAID pour la lutte contre le paludisme au titre de l’année fiscale 2015. Ouagadougou, Burkina Faso: USAID; 2015.
    6. Sirima SB, Konaté A, Tiono AB, Convelbo N, Cousens S, Pagnoni F. Early treatment of childhood fevers with pre‐packaged antimalarial drugs in the home reduces severe malaria morbidity in Burkina Faso. Trop Med Int Health. 2003;8:133-9.
    7. PMI VectorLink Project. 2019 Burkina Faso end of spray report: June 06, 2019 - July 11, 2019. Rockville, Maryland, USA: PMI VectorLink Project and Abt Associates Inc.; 2019.
    8. Sirima SB, Tiono AB, Konat AT, Badolo A, Traoré A, Apollinaire N, et al. Evaluation de la couverture de la population par les moustiquaires imprégnées d’insecticides après la campagne de distribution universelle en 2010. Ouagadougou, Burkina Faso: Ministère de la Santé; 2012.
    9. Programme National de Lutte contre le Paludisme. Rapport général de la campagne nationale de distribution universelle des MILDA au Burkina Faso en 2013. Ouagadougou, Burkina Faso: Ministère de la Santé; 2013.
    10. Institut National de la Statistique et de la Démographie (INSD), Programme d’Appui au Développement Sanitaire (PADS), Progamme National de Lutte contre le Paludisme (PNLP), ICF. Enquête sur les indicateurs du paludisme au Burkina Faso (2017-2018). Rockville, Maryland, USA: INSD, PADS, PNLP, and ICF; 2018.
    11. Ministère de la Santé. Annuaire statistique 2017. Ouagadougou, Burkina Faso: Conseil National de la Statistique; 2018.
    12. Badolo A, Traore A, Jones CM, Sanou A, Flood L, Guelbeogo WM, et al. Three years of insecticide resistance monitoring in *Anopheles gambiae* in Burkina Faso: resistance on the rise? Malar J. 2012;11:232.
    13. US President’s Malaria Initiative (PMI). Mozambique: malaria operational plan FY 2018. Bethesda, Maryland, USA: PMI; 2018.
    14. Central Intelligence Agency: The World Factbook Mozambique. [https://www.cia.gov/library/publications/the-world-factbook/geos/mz.html. Accessed 14 Jun 2019](https://www.cia.gov/library/publications/the-world-factbook/geos/mz.html.%20Accessed%2014%20Jun%202019).
    15. Food and Agriculture Organization of the United Nations (FAO): FAO in Mozambique. <http://www.fao.org/mozambique>. Accessed 28 June 2021.
    16. World Bank Open Data: Mozambique. <https://data.worldbank.org/indicator/SE.ADT.LITR.ZS?locations=MZ&view=chart>. Accessed 28 June 2021.
    17. United Nations, Department of Economic and Social Affairs, Population Division. World Population Prospects 2019. <https://population.un.org/wpp/>. Accessed 2 Jul 2021.
    18. National Malaria Elimination Programme (NMEP), National Population Commission (NPC), National Bureau of Statistics, ICF International. Nigeria Malaria Indicator Survey 2015. Abuja, Nigeria, and Rockville, Maryland, USA: NMEP, NPC, and ICF International; 2016.
    19. US President’s Malaria Initiative (PMI). Nigeria malaria operational plan FY 2019. Bethesda, Maryland, USA: PMI; 2018.
    20. National Population Commission (NPC), ICF. Nigeria Demographic and Health Survey 2018. Abuja, Nigeria, and Rockville, Maryland, USA: NPC and ICF; 2019.
    21. Central Intelligence Agency (CIA) The World Factbook: Rwanda. https://www.cia.gov/library/publications/the-world-factbook/. Accessed 17 April 2019.
    22. National Institute of Statistics of Rwanda (NISR). Fourth population and housing census – 2012. Kigali City, Rwanda: NISR; 2014.
    23. National Institute of Statistics of Rwanda (NISR), Ministry of Health (MOH) and ICF International. Rwanda Demographic and Health Survey 2014 – 15. Rockville, MD: NISR, MOH, and ICF International; 2015.
    24. Republic of Rwanda. Ministry of Health. 2015 Annual Health Statistics Booklet. Kigali, Rwanda; 2015.
    25. United Nations: Outreach programme on the 1994 genocide against the Tutsi in Rwanda and the United Nations. <https://www.un.org/en/preventgenocide/rwanda/>. Accessed 28 May 2020.
    26. US President’s Malaria Initiative (PMI). Rwanda malaria operational plan FY 2019. Bethesda, Maryland, USA: PMI; 2018.
